# Supplementary figures and images for: Plasticity of Drosophila germ granules during germ cell development
Source: PLoS Biol. 2023 Apr 13;21(4):e3002069. doi: 10.1371/journal.pbio.3002069 (PMC10128949; doi:10.1371/journal.pbio.3002069)

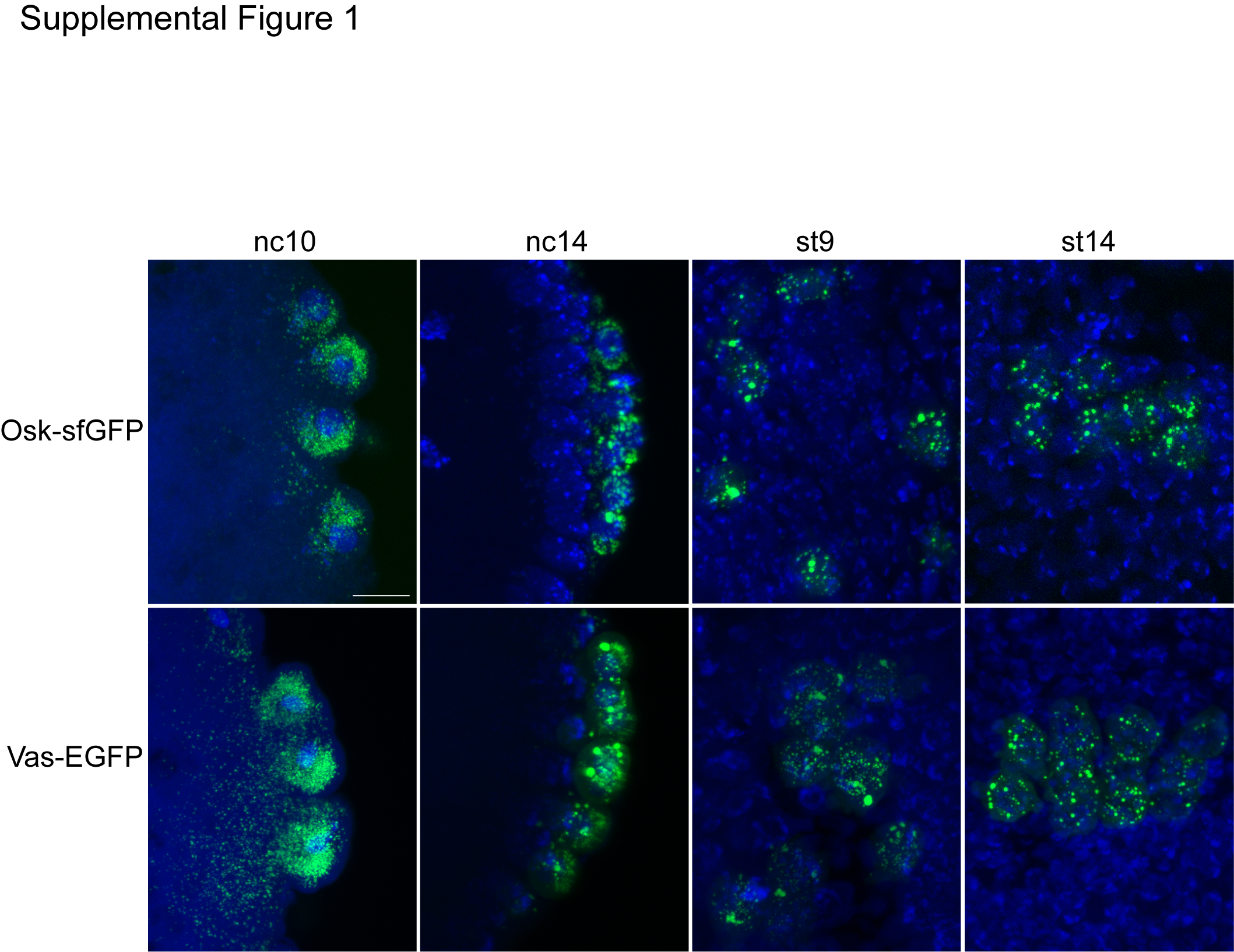

Supplement: S1 Fig — Maximum intensity confocal z-projections of representative pole cells at nc10, nc14, stage 9, and stage 14. Embryos were staged by nuclear cycle or Bownes stage according to nuclear density or morphological features, respectively. Osk-sfGFP (green) was visualized by anti-GFP immunofluorescence; Vas-EGFP (green) was detected by direct fluorescence; nuclei were stained with DAPI (blue). The brightness and contrast were adjusted individually for each image to best show the features of the germ granules at that stage. Scale bar: 10 μm. (TIF) [file pbio.3002069.s001.tif]

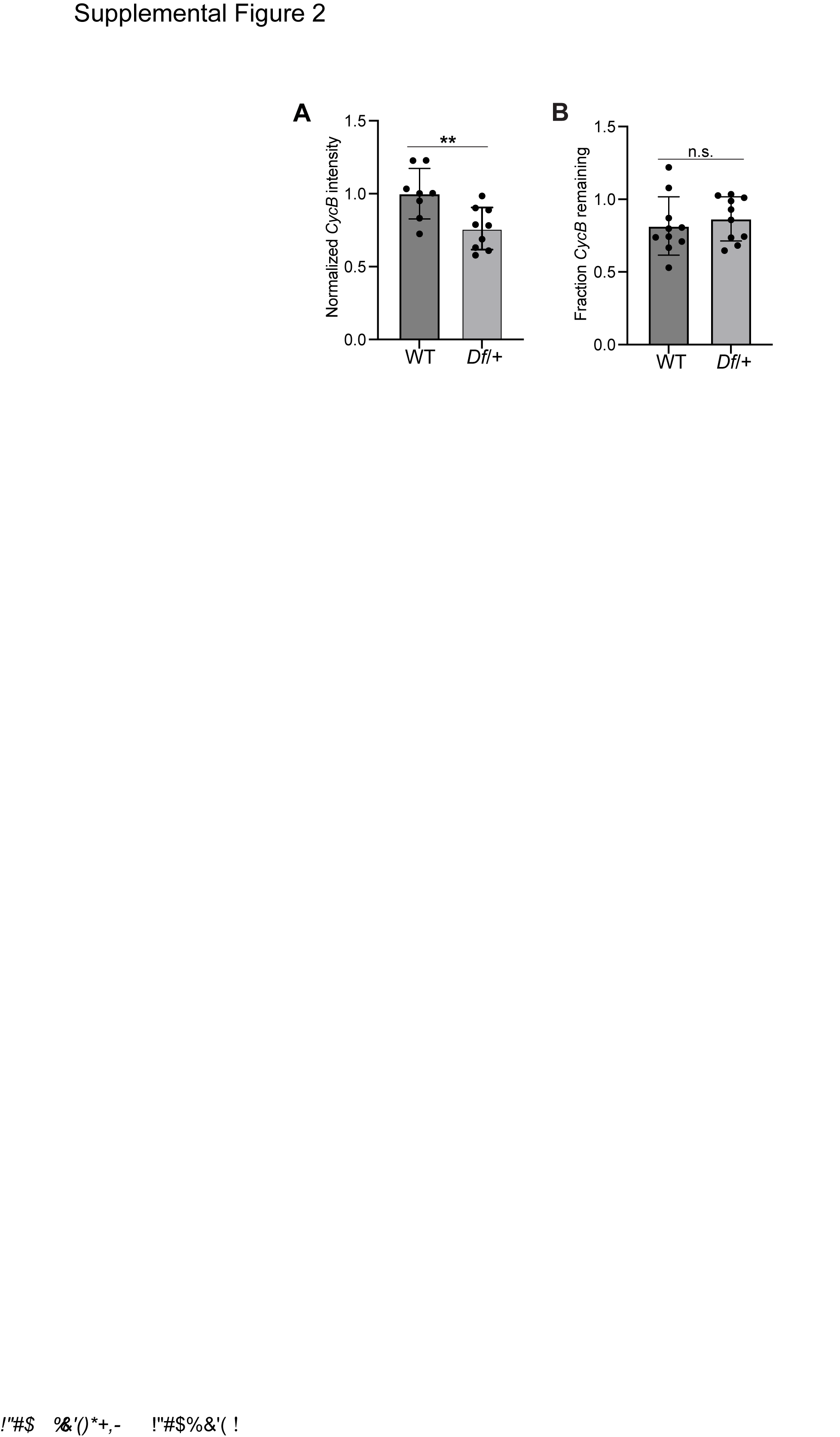

Supplement: S2 Fig — (A, B) CycB was detected by smFISH in wild-type embryos and in embryos heterozygous for a chromosomal deficiency (Df) that removes CycB. Total CycB intensity in the germ plasm was quantified at nc10-11 and at nc14. (A) Total CycB intensity at nc10-11 normalized to wild type, n = 8–11 embryos per genotype. (B) Total CycB intensity at nc14 normalized to the average nc10-11 intensity per genotype, n = 10 embryos. Graphs show individual data points and mean ± SD. **p < 0.001 and n.s., not significant, as determined by Student’s t test. Source data for the graphs in S2A and S2B Fig are provided in S1 Data. (TIF) [file pbio.3002069.s002.tif]

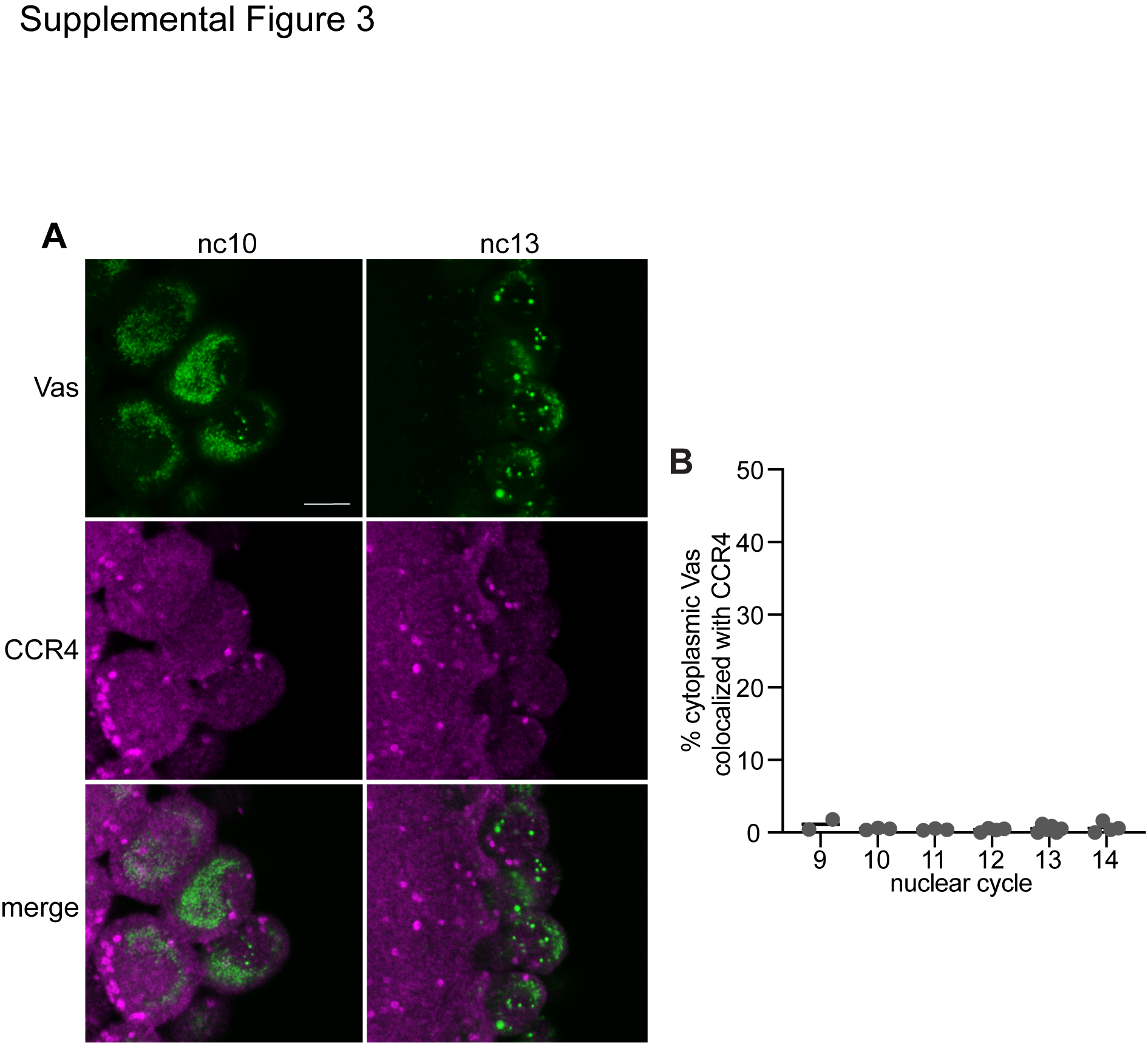

Supplement: S3 Fig — (A) Single confocal sections of the posterior region of representative syncytial blastoderm stage embryos expressing a vas-egfp transgene to mark the germ granules. Vas-EGFP was detected by direct fluorescence (green) together with anti-CCR4 immunofluorescence (magenta). (B) The percent of cytoplasmic Vas puncta that colocalize with CCR4 puncta was quantified at each nc, n = 2–6 embryos per nc. Nuclear Vas puncta were masked using Imaris software. Individual data points and means are displayed. Source data for the graph in S3B Fig are provided in S1 Data. (TIF) [file pbio.3002069.s003.tif]

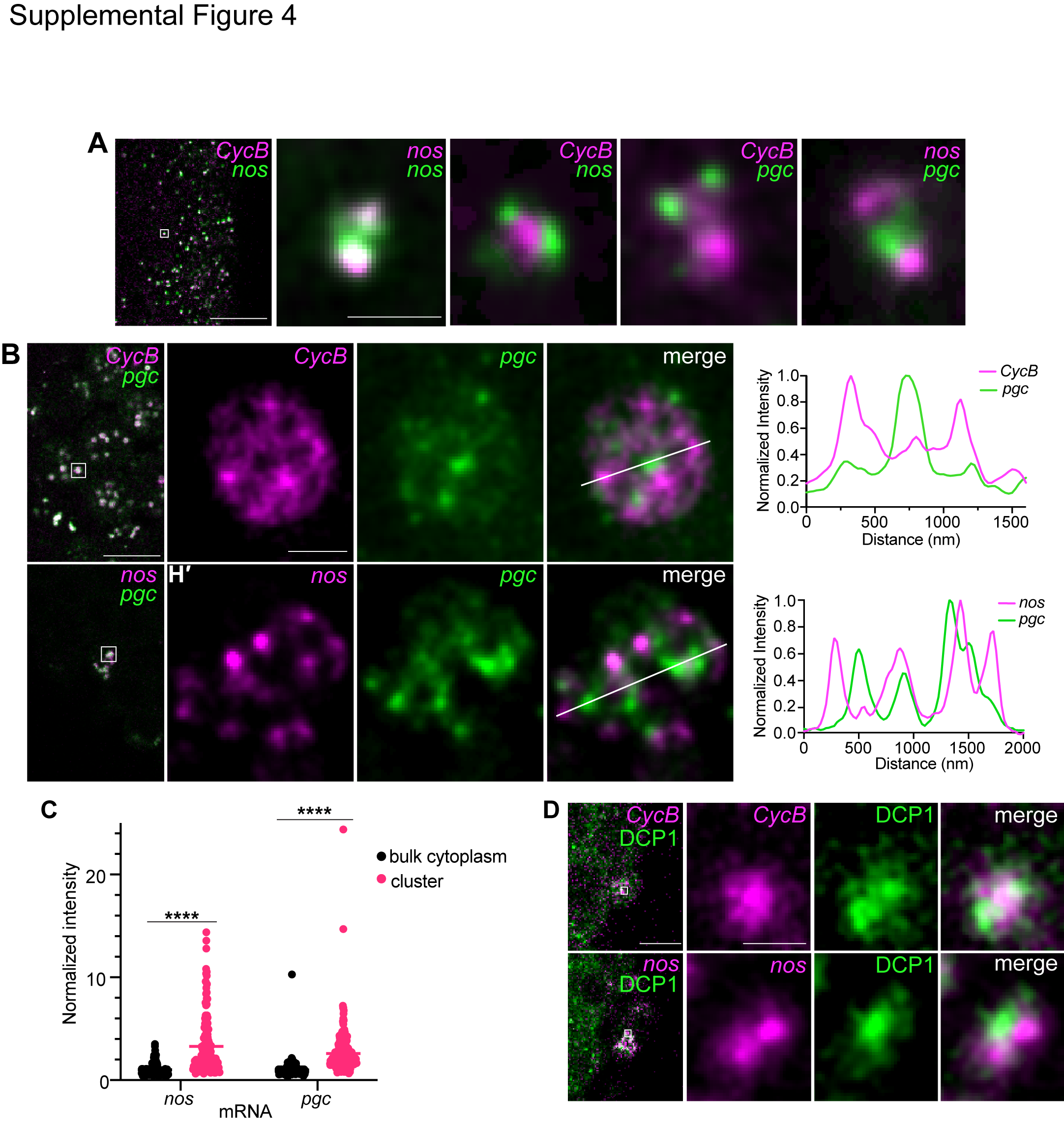

Supplement: S4 Fig — (A) The 2D STED images of nos, CycB, and pgc RNA detected pairwise by smFISH in granules in pre-pole bud stage embryos. Individual granules were selected for STED imaging from confocal images as shown in the example on the left (white box). (B) The 2D STED images (indicated by the white boxes on the confocal sections shown in the left-most panels) from pole cells at nc14. pgc (green) was detected together with CycB or nos (magenta) by smFISH. Fluorescence intensity was measured along the paths marked with white lines and intensity profiles of each channel, normalized to the maximum value, are plotted. (C) The sum intensity of nos and pgc puncta were measured from STED images of the bulk cytoplasm of early embryos (black data points) and of clusters in single germ granules (magenta data points). Values were normalized to the average intensity of a puncta in the bulk cytoplasm. Individual data points and means are displayed. ****p < 0.0001 by Mann–Whitney test. Source data for the graphs in S4B and S4C Fig are provided in S1 Data. (D) The 2D STED images (indicated by the white boxes on the confocal sections shown in the left-most panels) from pole cells in nc12 and nc13 embryos comparing the distribution of CycB or nos (magenta) to the distribution of DCP1 (green). RNAs were detected by smFISH and DCP1 was detected by immunofluorescence. Scale bars: 10 μm for confocal images; 500 nm for STED images. (TIF) [file pbio.3002069.s004.tif]

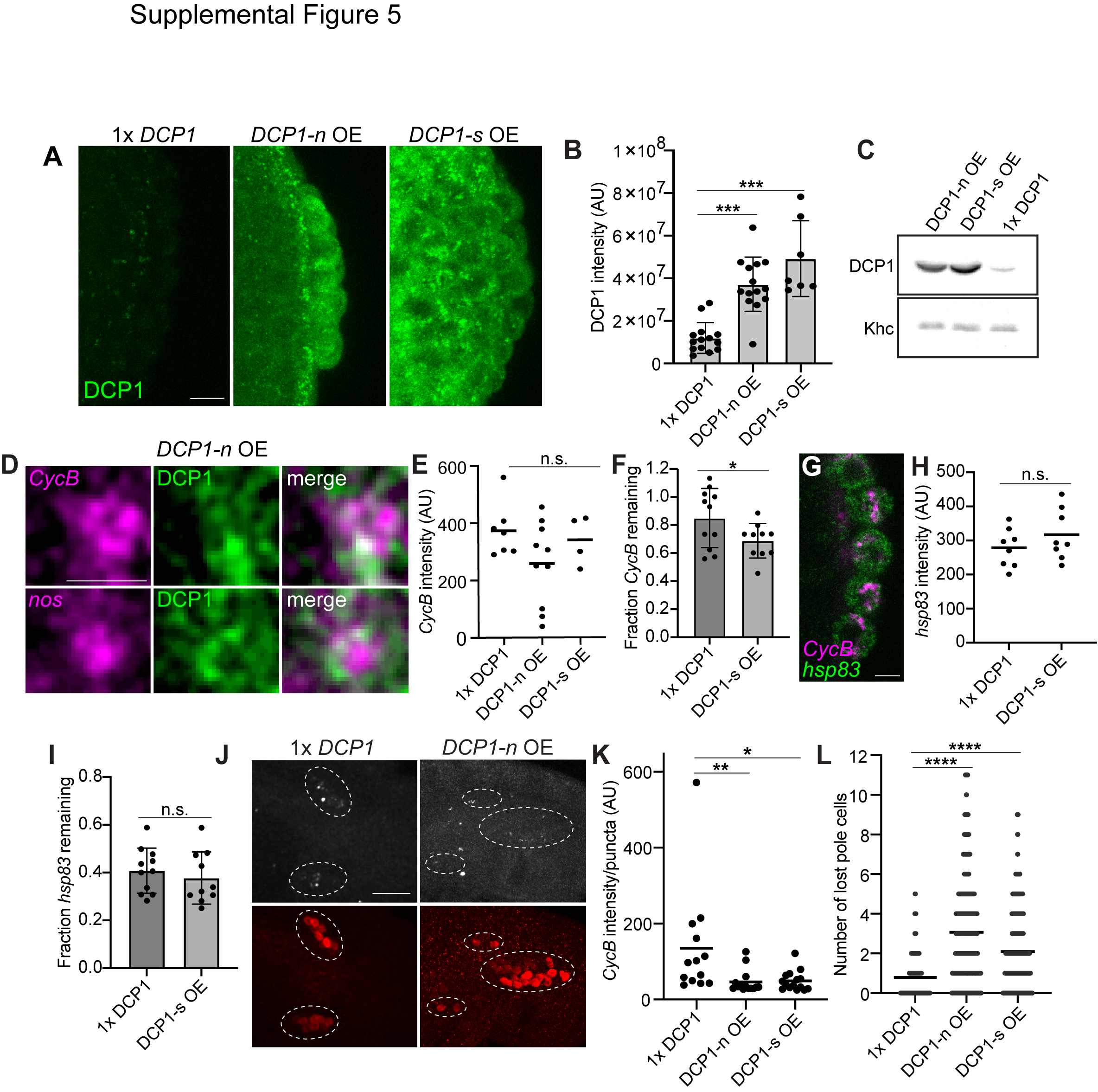

Supplement: S5 Fig — (A) Maximum intensity confocal z-projections of the posterior of syncytial blastoderm stage embryos that are heterozygous for a DCP1 mutation (1× DCP1) or have DCP1 overexpressed under control of the nos 3′UTR (DCP1-n) or smg 3′UTR (DCP1-s). DCP1 (green) was detected by immunofluorescence. (B) Quantification of the sum fluorescence intensity of DCP1 in the pole cells, n = 7–14 embryos per genotype. (C) Western blot analysis of DCP1 levels in embryos overexpressing DCP1 compared to DCP1 heterozygotes. Khc was used as a loading control. For the unprocessed data, see S1 Raw Images. (D) The 2D STED images of DCP1 (green) relative to CycB or nos (magenta) in DCP1-nos3′UTR overexpressing embryos. (E, F) CycB was detected by smFISH and the total CycB intensity in pole cells was quantified at nc10-13, n = 4–10 embryos per genotype (E) and nc14, n = 10–11 embryos per genotype (F). Total CycB intensity at nc14 was normalized to the average intensity at nc10-11 (F). (G) hsp83 (green) detected together with CycB (magenta) by smFISH in a wild-type nc14 embryo showing enrichment of hsp83 in pole cells, but not in germ granules. (H) Total hsp83 intensity in pole cells at nc10-11 (H). (I) Total hsp83 intensity at nc14 was normalized to the average intensity at nc10-11 for the same genotype, n = 10–11 embryos per genotype. (J) Maximum intensity confocal z-projections of CycB (gray) and Vas (red) in the gonads of DCP1 heterozygous and DCP1-nos3′UTR overexpressing embryos. CycB was detected by smFISH and Vas by immunofluorescence. (K) The average intensity of CycB puncta per embryo in DCP1 heterozygous, DCP1-nos3′UTR, and DCP1-smg3′UTR (also shown in Fig 5) overexpressing embryos was measured using Imaris, n = 13–15 embryos. (L) Quantification of the number of lost pole cells, detected by anti-Vas immunohistochemistry in DCP1 heterozygotes, DCP1-nos3′UTR, and DCP1-smg3′UTR (also shown in Fig 5) overexpression embryos, n = 73–223 embryos per genotype. Graphs display individual data poi [file pbio.3002069.s005.tif]

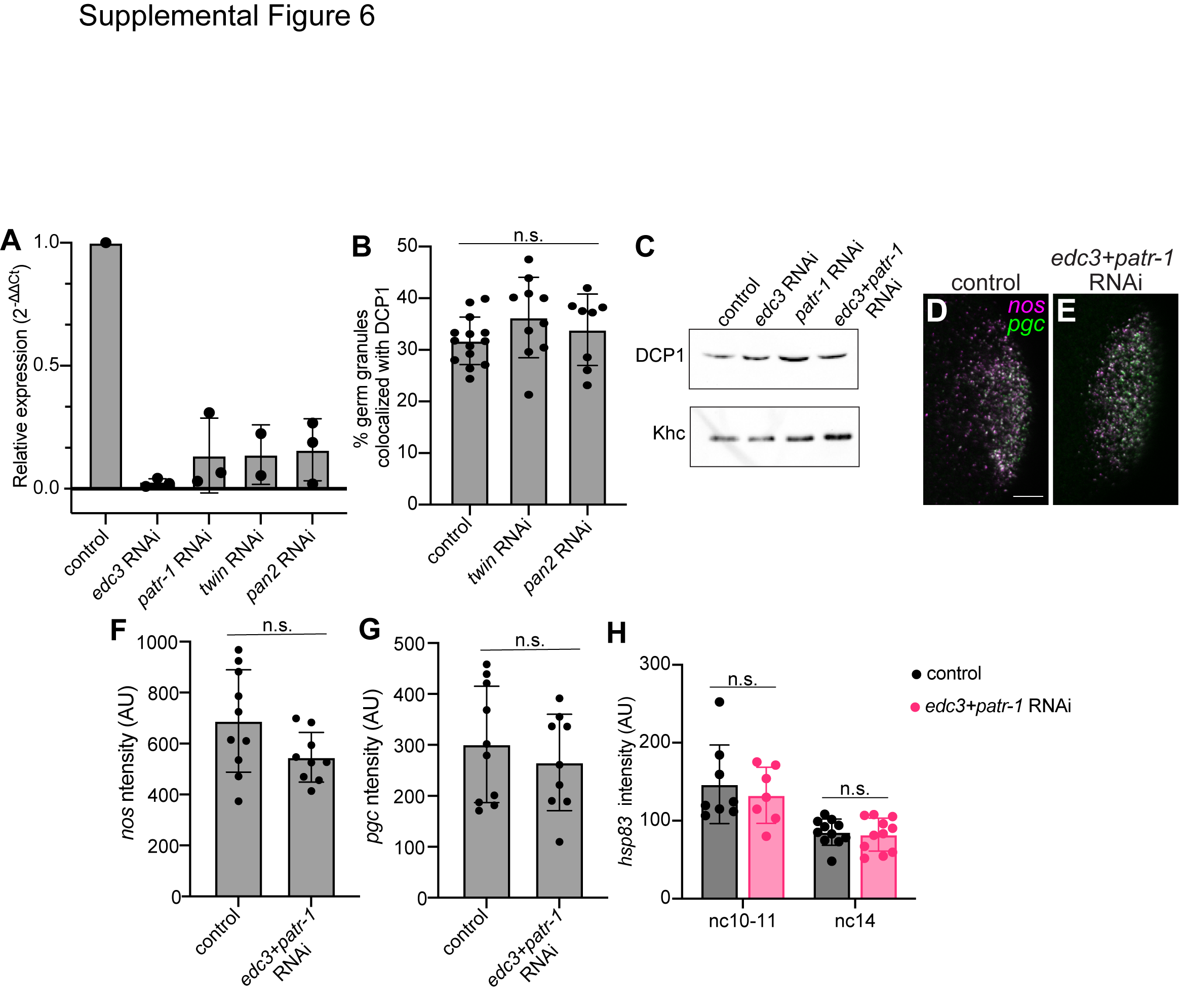

Supplement: S6 Fig — (A) RT-qPCR analysis of edc3, patr-1, twin, and pan2 RNA levels in 0–2 h embryos. The relative expression (2-ΔΔCT) of the target transcript for each RNAi was normalized to the expression in matα-GAL4 only controls. Data are averages of biological replicates. (B) Quantification of the percent of cytoplasmic Osk-GFP puncta that colocalize with DCP1 in control, twin RNAi, and pan2 RNAi embryos, n = 8–14 embryos per genotype. Genotypes are compared by Ordinary one-way ANOVA. (C) Western blot analysis of DCP1 levels in 0–2 h old matα-GAL4 only, edc3 RNAi, patr-1 RNAi, and edc3+patr-1 double RNAi embryos. Kinesin heavy chain (Khc) is used as a loading control. For the unprocessed data, see S1 Raw Images. (D, E) Confocal images of nos (magenta) and pgc (green) detected by smFISH in the germ plasm prior to pole cell budding in matα-GAL4 control (D) and edc3+patr-1 double RNAi (E) embryos. (F, G) Total nos (F) and pgc (G) intensities in the pole cells were quantified between nc9 and nc13, n = 9–10 embryos per genotype. (H) Total hsp83 intensity in the pole cells was quantified at nc10 and nc14, n = 7–11 embryos per genotype. RNA levels in matα-GAL4 only controls and edc3+patr-1 double RNAi embryos were compared by Mann–Whitney test. n.s., not significant. Individual data points and mean ± SD are shown. Source data for the graphs in S6A, S6B and S6F–S6H Fig are provided in S1 Data. (TIF) [file pbio.3002069.s006.tif]

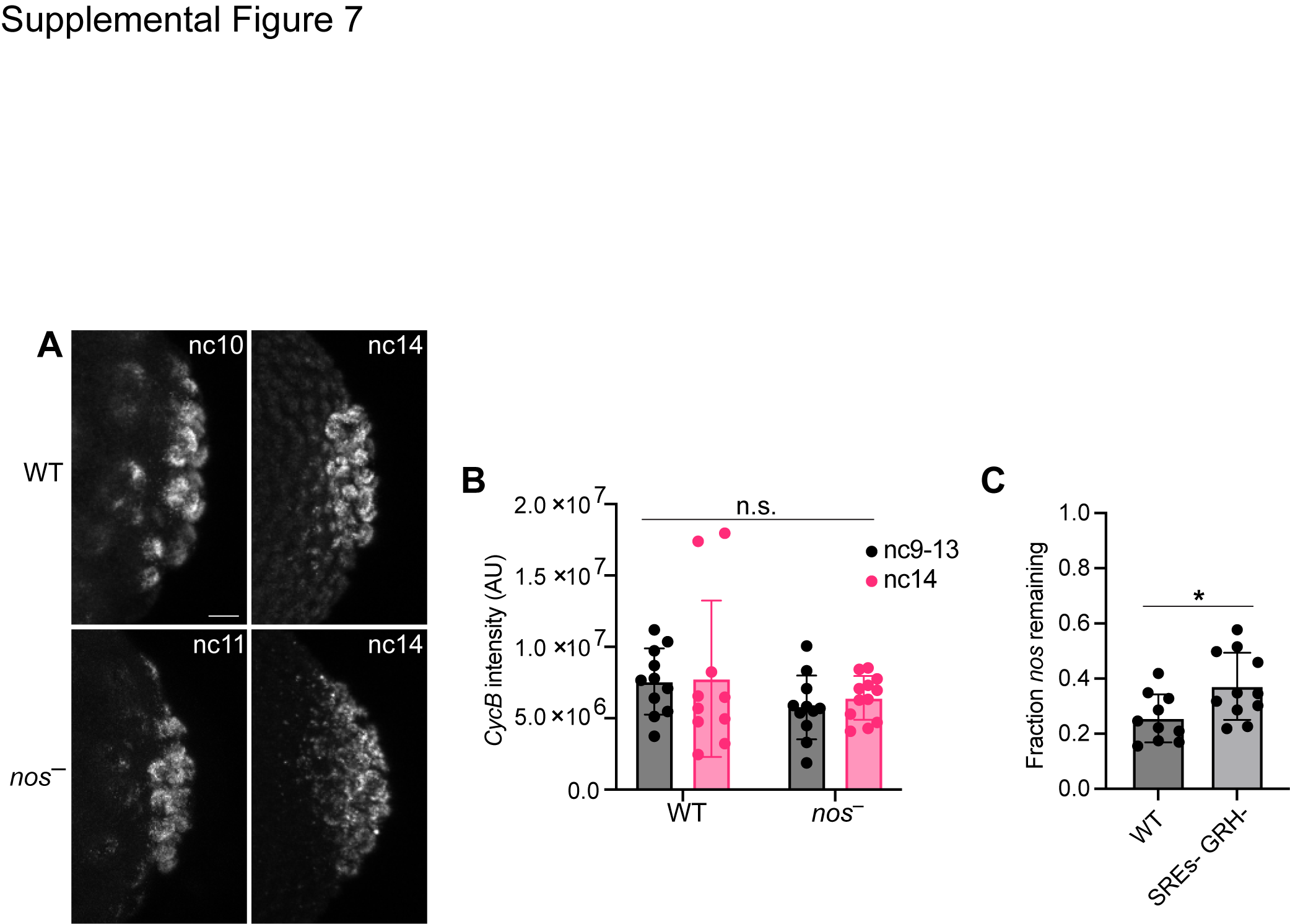

Supplement: S7 Fig — (A) Maximum intensity confocal z-projections of the posterior of wild-type (WT) and nos mutant (nos–) embryos. CycB was detected by smFISH during nc9-13 and at nc14. Representative embryos at nc10 (WT), nc11 (nos–), and nc14 (both) are shown. (B) Quantification of the sum fluorescence intensity of CycB in the germ plasm, n = 10–12 embryos per time point. CycB levels were compared by Kruskal–Wallis ANOVA. n.s., not significant. (C) nos was detected by smFISH in wild-type and gnosSREs-GRH- embryos at nc10-11 and nc14. The gnosSREs-GRH- transgene produces nos mRNA with mutations in binding sites for the Smaug (SREs) and Glorund (GRH) repressors (51). Total nos fluorescence intensity in the germ plasm was quantified and the intensity at nc14 was normalized to the average intensity at nc10-11, n = 10–11 embryos per genotype. *p < 0.05 by Student’s t test. Individual data points and means ± SD are shown. Source data for the graphs in S7B and S7C Fig are provided in S1 Data. Scale bar: 10 μm. (TIF) [file pbio.3002069.s007.tif]

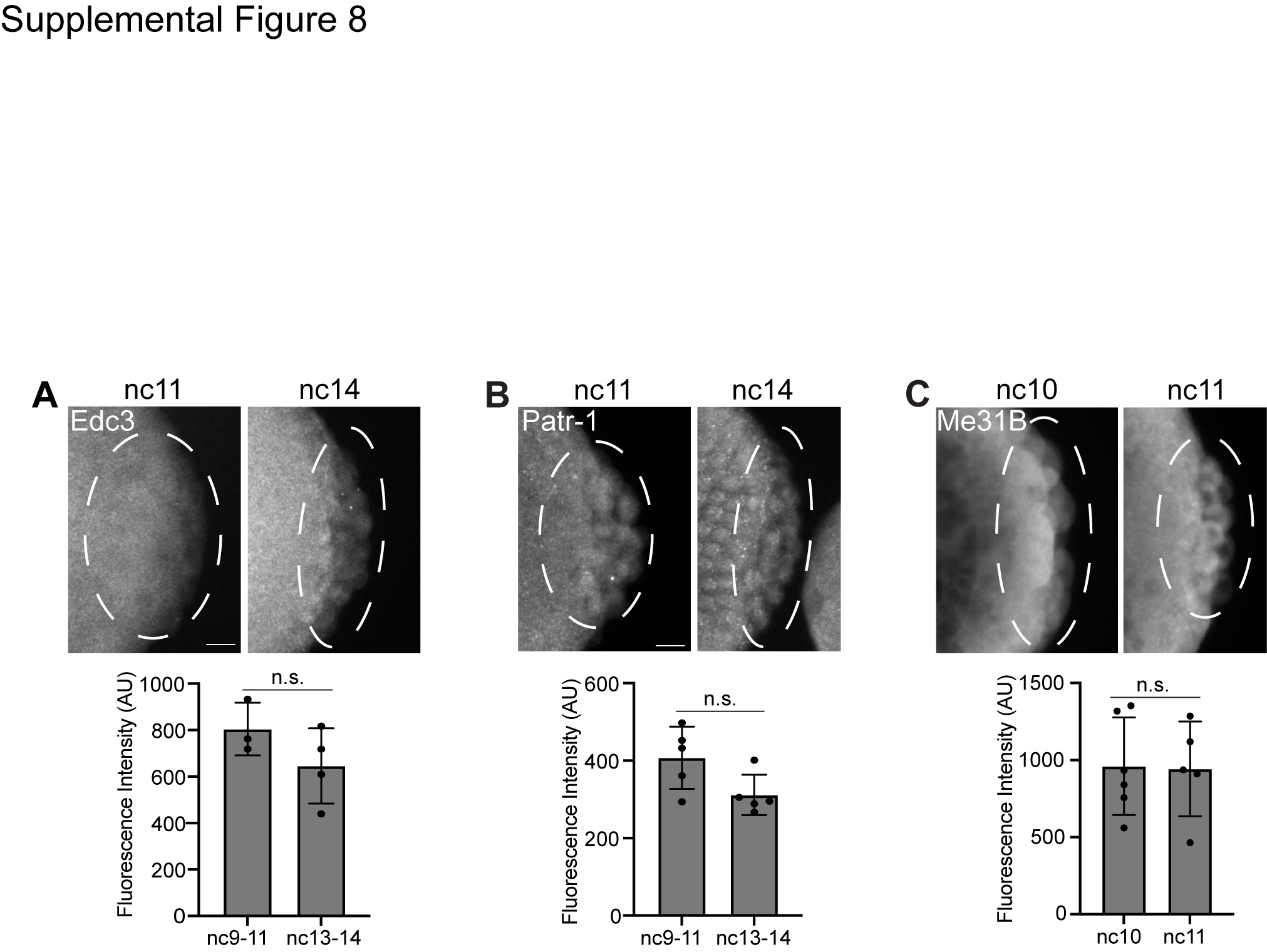

Supplement: S8 Fig — (A–C) Sum intensity confocal z-projections of the posterior of nc10, nc11, and nc14 embryos. Edc3 (A), Patr-1 (B), and Me31B-GFP (C) were detected by immunofluorescence. White circles indicate the region of the pole cells. Total fluorescence intensity in the pole cell region of embryos during nc9-11 and nc13-14 (A, B) or nc10 and nc11 (C) was quantified, n = 3–6 embryos each. n.s., not significant by Student’s t test. Source data for the graphs in S8A–S8C Fig are provided in S1 Data. Scale bar: 10 μm. (TIF) [file pbio.3002069.s008.tif]
