# Supplementary material for: Plasticity of Drosophila germ granules during germ cell development
Source: PLoS Biol. 2023 Apr 13;21(4):e3002069. doi: 10.1371/journal.pbio.3002069 (PMC10128949; doi:10.1371/journal.pbio.3002069)
Supplement: S1 Raw Images — TIF images of raw blots uncropped and labeled with antibody used and sample identifier. Lanes indicated by X’s are not relevant to Figs 8B and S5C. (PDF) [file pbio.3002069.s009.pdf]

Fig 8B Uncropped Western Blot

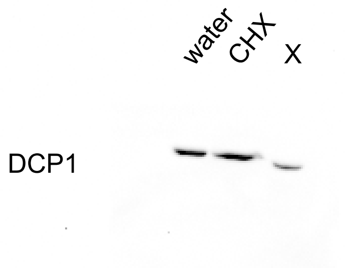

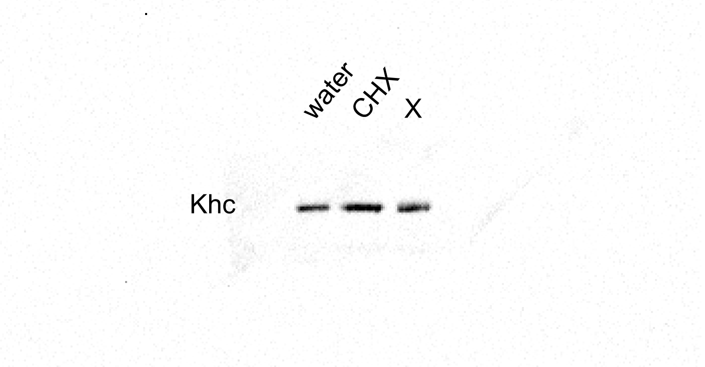

Lanes not included in Fig 8 are marked as X

S5C Fig Uncropped Western Blot

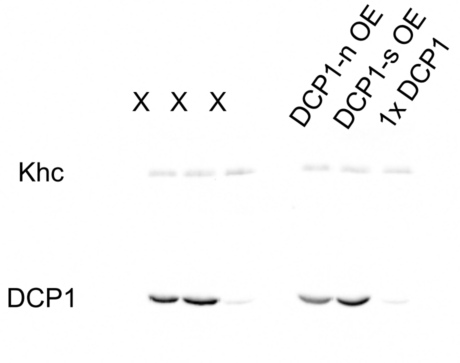

Lanes not included in S5 Fig are marked as X

S6C Fig Uncropped Western Blot

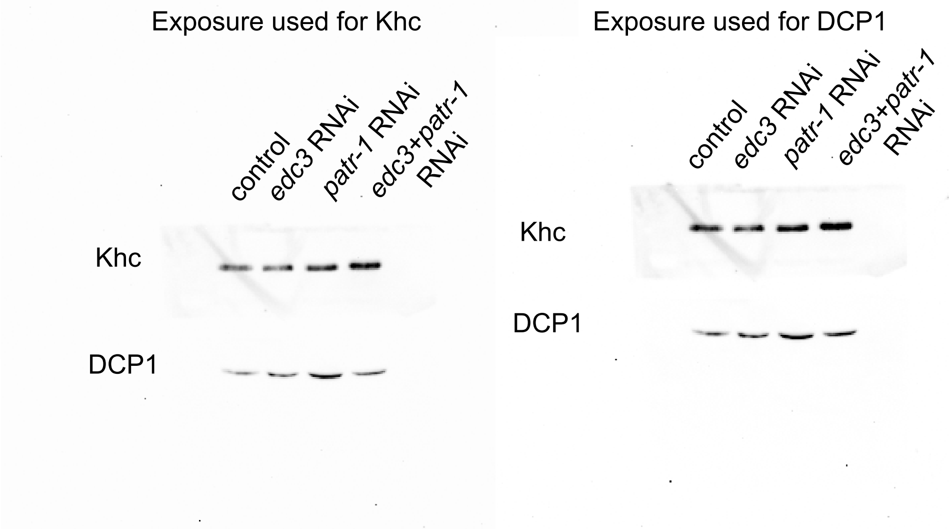

All images were captured using iBright FL 1000 Imaging System
