# Supplementary material for: Plasticity of Drosophila germ granules during germ cell development
Source: PLoS Biol. 2023 Apr 13;21(4):e3002069. doi: 10.1371/journal.pbio.3002069 (PMC10128949; doi:10.1371/journal.pbio.3002069)
Supplement: Attachment — Submitted filename: Response to Reviewers.dotx [file pbio.3002069.s014.dotx]

**Response to Reviewers**

We are glad that the reviewers are enthusiastic about our work. We thank them for their thoughtful reviews and helpful suggestions. We have done our best to address all of the criticisms through the addition of new experiments and modifications to the text as outlined in detail below. We believe the additional data and discussion have greatly improved the manuscript and strengthened our conclusions.

**Reviewer 1**

*1. Selective degradation of germ granule mRNAs: The authors observe that nos and pgc mRNAs start to be degraded during pole cell formation, whereas the CycB transcript is stable. According to RNA-seq data in the Flybase, however, the amount of CycB mRNA in 0-2 h embryos is about five-fold compared with those of nos and pgc. My personal experience of in situ hybridization with the AP-conjugated anti-DIG antibody followed by X-phosphate/NBT staining supports that CycB is the most abundant germ plasm mRNA. If the speed of degradation (e.g. molecules/time) is constant among germ granule mRNAs, the rate of decrease in the CycB mRNA level would be much slower. For example, when nos or pgc mRNA levels decreased to 5%, still >80% CycB mRNA would remain. If this is the case, there would be no selective mechanism for the degradation of germ granule mRNAs*

*CycB* is the most abundant germ granule RNA, but RNA-seq data from 0-2 hour embryos is dominated by the soma, which makes up most of the volume of the embryo and contains the vast majority of the *CycB*, *nos*, and *pgc* transcripts. Recent smFISH analysis that measured mRNA levels specifically in the germ plasm of early embryos found that the level of *CycB* is 1.8-fold higher than *nos* (Trcek et al. 2020 doi.org/10.1016/j.molcel.2020.05.008; Fig. 1 and Table S1). We have added data in Figure 3K-L showing that increasing the initial amount of *nos* in the pole cells by 1.6-fold (to more closely match CycB) using a *nos* transgene did not alter the extent of *nos* degradation at nc14. We also added a new Supplementary Figure (S2 Fig) showing that decreasing the initial amount of *CycB* in embryos heterozygous for a *CycB* deficiency does not alter *CycB* stability. We believe that these data greatly strength our conclusion that there is differential regulation of RNA degradation.

2. *Physiological roles of selective degradation of germ granules mRNAs: It is plausible that, when a sufficient amount of nos mRNA is localized in the germ plasm, ~20% nos mRNA in stage-5 (nc14) pole cells would be enough for its function during subsequent embryogenesis (the reduction of nos and pgc mRNAs in pole cells is a physiologically normal event during their removal in the somatic region). If the degradation mechanism is turned not to be selective, I think that the authors will have to dramatically rebuild the overall logical flow in this manuscript.*

We have provided further evidence that the degradation is selective (see #1). We agree that 20% of *nos* mRNA appears to be sufficient as Nos protein is found in the pole cells throughout embryogenesis, which we mention briefly in the discussion (lines 457-459).

3. *How about PARN? In vertebrates, PARN, which is another type of deadenylase, forms a complex with CPEB (the Drosophila Orb homolog) and GLD2, which is a cytoplasmic poly(A) polymerase. The competition between PARN and GLD2 in the CPEB complex regulates the poly(A) tail length of Cyclin-B1 mRNA in the Xenopus oocyte (Kim and Richter, Mol. Cell 2006). Although the distribution of Orb protein in early-stage embryos has not been documented at a high resolution, orb mRNA is known to be enriched in the germ plasm (Lécuyer et al., Cell 2007). The authors may be worthy of examining the localization of Orb complex during pole cell formation.*

PARN is found only in vertebrates and therefore does not have a *Drosophila* ortholog. However, the reviewer raises a good point that other deadenylases could be involved. We therefore knocked down Pan2, the catalytic component of the other deadenylase complex in *Drosophila*. Like *twin* RNAi, DCP1 recruitment is not affected in *pan2* RNAi embryos, providing further evidence that deadenylation is not required for DCP1 recruitment. These data have been added to panels S6A-B.

*Also, the poly(A) tail is often shortened in a translation-dependent manner, which triggers decapping and mRNA decay (Funakoshi et al. Genes Dev 2007).*

We had indeed tested the hypothesis that increased translation could trigger degradation of transcripts by analyzing *CycB* in *nos* mutants (shown in the original S6B and S6C Fig, now S7B and S7C Fig). We have modified the text describing this experiment to better explain the relationship between increased translation and shorter polyA tail length (lines 337-339).To complement this analysis, we monitored the stability of a translationally derepressed *nos* RNA that produces excess Nos protein. This *nos* RNA is not more efficiently degraded than wild-type *nos*, further supporting the conclusion that increased translation does not enhance mRNA decay in germ granules. The new data are shown in S7D Fig.

4. *DCP1 overexpression and edc3/patr1 RNAi: The authors show that the overexpressed DCP1 accumulates in the germ granules and causes CycB mRNA degradation. Fig S4 shows that DCP1 is also present in the cytoplasm when it is overexpressed. I wonder if the overexpression of DCP1 also promotes the degradation of another class of pole cell mRNAs such as wun2 and tre1 mRNAs, which appear not to reside in the germ granules but play important roles in steering pole cell migration (Kunwar et al., PLOS Biol. 2003; Hanyu-Nakamura et al. Development 2004; Renault et al., Science 2004). A similar question is also to the edc3/patr1 RNAi, which reduces DCP1 association to the germ granules (Fig 6). Because the edc3/patr1 RNAi does not affect the total DCP1 level, the cytoplasmic DCP1 level is likely to be increased. If these non-granular mRNAs are degraded by DCP1 overexpression and edc3/patr1 RNAi, another hypothesis would be that DCP1 has to be sequestered in the separate compartments within the germ granules for the protection of maternal mRNAs in pole cells.*

We thank the reviewer for this interesting alternate hypothesis. smFISH analysis shows that *wun2* and *tre1* are found in the germ granules (R. Lehmann, personal communication to E.R.G.) and their relatively low levels in the germ plasm would make detection of degradation difficult. Therefore, to address the question of whether the stability of non-granule RNAs in the pole cells are affected by these manipulations, we evaluated *hsp83*, an abundant mRNA that is located outside of germ granules. In wild-type embryos, *hsp83* levels decrease by about 60% between nc 10-11 and nc14. Degradation of *hsp83* is not significantly affected by either DCP1 overexpression or *edc3/patr-1* RNAi. These data have been added to S5G-HI Fig and S6H Fig respectively.

*5. Defects in pole cell migration: The authors show that DCP1 overexpression (enhancement of decapping) and edc3/patr1 RNAi (diminishment of decapping) both cause defects in pole cell migration. How do the authors interpret the results from the opposite manipulations?*

We believe that together, these results speak to the need for differential regulation – overexpression of DCP1 likely causes untoward decapping and degradation of RNAs encoding proteins needed for further germ cell development and function whereas *edc3/patr1 RNAi* results in stabilization of RNAs and production of proteins whose functions are no longer required and may inhibit further development. For example, Pgc suppresses the MZT in pole cells so destruction of *pgc* RNA would prevent unwanted Pgc synthesis. This interpretation is consistent with our evidence for selective degradation (see #1) and that non-granule RNAs are not widely mis-regulated with these manipulations (see #4) We now explain our interpretation of these findings in the Discussion section (page 18).

*6. Another question for these experiments is whether the germ plasm behaviors (fusion and enlargement) are affected by DCP1 overexpression and edc3/patr1 RNAi.*

Enlargement of germ granules does appear to be hindered by *edc3/patr-1* RNAi, as granules tend to be smaller at nc14 than controls. This observation is now mentioned in the results (lines 296-297). Growth of the germ granules does not appear to be affected in DCP1 overexpression embryos, which can be seen by comparing the STED images shown in Fig 5.

Minor points

*1. DCP1 is a co-activator of decapping catalyzed by the DCP2 subunit. The authors may need to examine the distribution of DCP2.*

Unfortunately, we were unable to perform this experiment because despite our best effort, we were unable to obtain an antibody to *Drosophila* DCP2*.* We attempted to visualize DCP2 using an antibody against human DCP2, which was used in *Drosophila* in a biorxiv paper (Kunar and Roy, 2019), but the sensitivity of this antibody was too low in the early embryo.

*2. The DCP1 signals are still detectable in somatic cells. Thus, I think that the authors still cannot exclude the possibility that DCP1 in the soma indirectly affects pole cell migration.*

We have added a sentence to acknowledge this possibility (lines 276-277).

*3. Page 12 and Fig 7 (nuclear Me31B signals): the signals are not clear in the figure. Please point the nuclear signals with arrowheads.*

We have added arrows to Fig 7A to indicate this Me31B signal

*4. Edc3 and Patr1 are known to interact with Me31B, which is a component of the osk RNP during oogenesis (ref. 44). Is the germ plasm assembly normal in edc3/patr1 RNAi embryos?*

Yes, germ plasm assembly is normal in these knockdowns, as evidenced by normal levels of *nos* and *pgc* in pole cells. We have also added images of pre-pole bud embryos in S6D-E Fig to provide additional evidence for that germ plasm assembly is unaffected.

**Reviewer 2**

*…molecular and biochemical analyses from Drosophila germ granules are not technically practical. However the work could be usefully extended by building on what has been found in yeast, where more is known about mechanisms of establishing selective degradation. Ref. 54 discusses how several different decapping activators bind to different sites in the C-terminal region of DCP2 and form different decapping complexes, which target different subsets of mRNAs.*

As suggested, we have expanded on our discussion of results from Ref. 54 and others that studied the specific molecular interactions within the decapping complex in yeast, as well as in *Drosophila*, and how these relate to our findings (lines 395-399 and lines 422-433).

*Are the binding sites for decapping activators conserved in fly DCP2?*

The binding sites for decapping activators are not conserved in fly DCP2. Overall, the C-terminal domain of DCP2 is not highly conserved and the C-terminus is truncated in *Drosophila* as compared to yeast, which eliminates most of these binding sites. We have now considered this lack of conservation in our discussion of how our findings expand on work in yeast (see above).

*While orthologs of some of these activators were included in this study, others (Upf1, Lsm1, tral (the Scd6 ortholog) were not. Nor was DCP2 analyzed in detail. Is it at least known whether the other decapping activators are present in germ plasm and therefore could contribute to specificity?*

As explained in our response to Reviewer 1 above, we were unable to obtain an antibody to *Drosophila* DCP2 and an antibody to human DCP2 was not effective in early embryos. To our knowledge, only the localization of Trailer Hitch (the Scd6 homolog) has been evaluated relative to the germ plasm. In oocytes and early embryos TraI localizes to the entire cortex, which would include a region of overlap with the germ plasm at the posterior (ref 44). We have added an acknowledgement that other decapping activators may contribute to DCP1 recruitment and specificity of mRNA degradation in the discussion (lines 422-433).

*Referring to the manuscript at the end of page 8 and beginning of page 9, the distributions of DCP1 and Pcm are described as similar to that of CCR4. I think the distributions are only similar in the sense that they form puncta that do not overlap with germ granules. Lacking co-localization experiments with CCR4 I think the sentence requires clarification.*

We did not intend to suggest DCP1 and Pcm colocalize with CCR4. We have amended the sentence to remove the comparison to CCR4 (lines 207-209)

*Line 14 of the abstract: should read 'differentially' not 'differential'.*

*On page 4, the Su et al. 1998 reference needs to be numbered (I think it is ref. 61).*

*On page 16, line 5, it should read 'repress' not 'represses'.*

We appreciate the reviewer catching these errors and have corrected them.

**Reviewer 3**

*Fig. 6A: the assembly of large cytoplasmic Osk granules appears to be disrupted by both edc3 and patr-1 RNAi; in the close-ups, cytoplasmic germ granules of RNAi strains are smaller than the control granules at the same stage. Instead of a failure of DCP1 localization to appropriately enlarged germ granules, it seems as though disruption of decapping prevents the developmental switch in germ granule morphology and function. This possibility should at least be acknowledged.*

As explained above in response to Reviewer 1, there does appear to an effect of *edc3+patr-1* RNAi on the enlargement of germ granules, which could indicate that decapping is required for their enlargement. We have added this observation and its implications to the text (lines 296-298).

*"Recruitment of DCP1 to germ granules is necessary for proper pole cell development":*

*The title of this section of the Results is problematic: The data does not distinguish whether the defect in edc3/patr-1 double RNAi is in fact DCP1's localization to germ granules versus DCP1's ability to be activated for decapping or to be targeted to specific mRNAs. If the embryo doesn't have patr-1/edc3, decapping will be compromised whether or not DCP1 is in the germ granules. The section title should be changed to avoid overinterpreting the results.*

We have changed the title of this section to remove this claim (line 355).
